# Direct Growth of Two Dimensional Molybdenum Disulfide on Flexible Ceramic Substrate

**DOI:** 10.3390/nano9101456

**Published:** 2019-10-14

**Authors:** Yixiong Zheng, Chunyan Yuan, Sichen Wei, Hyun Kim, Fei Yao, Jung-Hun Seo

**Affiliations:** 1 Department of Materials Design and Innovation, University at Buffalo, Buffalo, NY 14260, USA; yixiongz@buffalo.edu (Y.Z.); chunyany@buffalo.edu (C.Y.); sichenwe@buffalo.edu (S.W.); 2 Component Solution Business Unit, Samsung Electro-Mechanics, Suwon 16674, Korea; withblack23@gmail.com

**Keywords:** two-dimensional molybdenum disulfide, direct growth, flexible Yttria-stabilized zirconia substrate

## Abstract

In this paper, we report the first successful demonstration of the direct growth of high-quality two-dimensional (2D) MoS_2_ semiconductors on a flexible substrate using a 25-μm-thick Yttria-stabilized zirconia ceramic substrate. Few-layered MoS_2_ crystals grown at 800 °C showed a uniform crystal size of approximately 50 μm, which consisted of about 10 MoS_2_ layers. MoS_2_ crystals were characterized using energy-dispersive X-ray spectroscopy. Raman spectroscopy was performed to investigate the crystal quality under bending conditions. The Raman mapping revealed a good uniformity with a stable chemical composition of the MoS_2_ crystals. Our approach offers a simple and effective route to realize various flexible electronics based on MoS_2_. Our approach can be applied for MoS_2_ growth and for other 2D materials. Therefore, it offers a new opportunity that allows us to demonstrate high-performance flexible electronic/optoelectronic applications in a less expensive, simpler, and faster manner without sacrificing the intrinsic performance of 2D materials.

## 1. Introduction

Two-dimensional (2D) materials such as graphene, transition metal dichalcogenide, and X-enes including silicene, germanene, and phosphorene have emerged as a new class of materials for flexible electronics because of their exceptionally stable and robust electrical properties under mechanical strain conditions [1,2,3,4,5,6,7,8,9,10]. Graphene, for example, has a very high strain tolerance, which can be up to 40%; this is nearly an order of magnitude higher than other low dimensional inorganic crystalline materials such as silicon nanomembrane [11,12,13]. Under bending conditions, phosphorene exhibits a wide bandgap tunability range of about 1 eV with direct bandgap properties [14,15]. Such exceptional mechanical properties of 2D materials are primarily the result of atomic thinness and a low defect level, as well as a strong horizontal direction bonding force. Thus, 2D materials became the most promising material candidates for future flexible electronics such as gas sensors, photodetectors, transistors, wearable devices, and communication systems. [16,17,18,19] While these flexible electronics demonstrated superior mechanical properties, their electrical performance substantially decreased compared with the rigid version of the devices. For example, a rigid version of graphene microwave transistors can be operated up to 400 GHz [20], but a flexible version of graphene transistors only can operate up to 32 GHz [21], which is similar to or lower than other nanomaterial-based flexible electronics. The threshold voltage and subthreshold voltage swing of flexible MoS_2_ transistors are 2.1 V and 250 mV, respectively [22], which are much worse than their bulk counterparts [23].

This performance degradation is mainly caused by additionally introduced defects and unwanted molecules on the surface of 2D materials [24,25]. However, it is inevitable to avoid this degradation because it is required to create freestanding forms of 2D materials in order to transfer them onto a flexible substrate such as poly-ethylene terephthalate (PET) or polyimide (PI) substrates. Several routes such as the wet-etching and scooping method or the mechanical exfoliation method (also known as the Scotch tape method) have been commonly used to obtain 2D materials from their bulk source materials [26]. The Scotch tape method uses standard Scotch tape to remove 2D materials from bulk source materials. The tape is repeatedly folded and unfolded to exfoliate 2D materials, which are then transferred to a flexible substrate. This method enables us to produce high-quality graphene layers but obtained 2D materials are typically small, have unpredictable sizes, and are often difficult to reproduce. The wet-etching-and-scooping method normally uses 2D semiconductors that are grown via chemical vapor deposition (CVD) technique. CVD method has been proven to be a deterministic approach in terms of the large-scale production of 2D semiconductors with high quality and low manufacturing costs [27]. For MoS_2_, to be more specific, the growth can be divided into vapor sulfurization and vapor deposition depending on the method of introducing the source materials in the furnace. In the vapor sulfurization method, Mo precursors e.g., Mo or MoO_3_ are pre-deposited on the substrate, followed by sulphurization [28,29,30,31]. In contrast, in the vapor deposition method, the source materials such as MoO_3_ or MoCl_5_ are evaporated during the growth process [32,33,34]. Both of these two CVD methods require high temperature (up to ~800 °C) operation, which restricts the substrate selection to high-temperature tolerance materials [35]. Once 2D semiconductor layers are grown, they will then be separated from substrate using an etchant and then floating on a water surface. The obtained 2D materials are then directly scooped from the water surface to a flexible substrate. Because this method involves various polymers and chemicals, the surface of 2D materials has unwanted contamination caused by excessive numbers of chemical bonds from the polymer and etchant, which behave as scattering sites [36,37,38,39]. In fact, all of these issues can be solved by employing a high thermal tolerance flexible substrate, which can withstand the temperature requirement for the chemical vapor deposition (CVD) growth and thus enables us to grow 2D materials directly on the flexible substrate. Then, the benefits of 2D materials, such as the superior electrical properties with robust mechanical properties, can be fully utilized and potentially reduce or eliminate a performance degradation in 2D materials on a flexible substrate.

In this paper, we used a 25 μm thick Yttria (Y_2_O_3_)-stabilized zirconia (YSZ) ceramic [40] as a substrate for a 2D materials growth and successfully grew few-layered molybdenum disulfide (MoS_2_) directly on flexible YSZ substrate. To evaluate the crystal quality of directly grown MoS_2_, we have performed a quantitative study to evaluate the quality of MoS_2_ under bending conditions. Raman spectroscopy study revealed that the directly grown MoS_2_ crystals have good crystal uniformity and chemical composition. Furthermore, a Raman shifting rate of the E^1^_2g_ and A_1g_ peaks in MoS_2_ crystals under strain conditions is in good agreement with other results [41], suggesting that the quality of directly grown MoS_2_ crystals is similarly good compared to MoS_2_ crystals that are grown on a rigid substrate.

## 2. Materials and Methods

**Figure 1:**
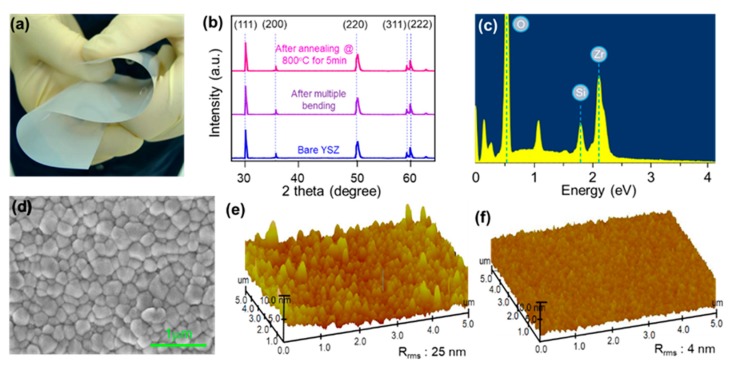
(**a**) a bent image of flexible Yttria-stabilized zirconia ceramic (YZS) substrate, (**b**), X-ray diffraction (XRD) spectra of a YZS substrate (red) a bare condition, (orange) after 20 times of bending, (blue) after the annealing at 800 °C for 5 min. (**c**) energy-dispersive X-ray spectroscopy (EDX) spectrum and (**d**) secondary electron microscopy (SEM) image of SiO_2_ coated YZS substrate. Atomic force microscopy (AFM) images of (**e**) a bare YZS substrate, (**f**) a 50 nm thick SiO_2_ coated YZS substrate.

A YSZ is a ceramic that has received a lot of attention due to its exceptional thermo-mechanical properties such as a high degree of hardness, a high dielectric constant, chemical inertness, and a high ionic conductivity at elevated temperatures. As shown in Figure 1a, we have employed 25 μm thick flexible YSZ substrates (3 mol% Y_2_O_3_-stabilized tetragonal zirconia ceramic produced by ENrG Inc. (Buffalo, NY, USA) as a flexible substrate for direct MoS_2_ growth [40]. As shown in Figure 1b, Rigaku D/max-2500 diffractometer with Cu Kα radiation (λ = 1.5418) was used for X-ray diffraction (XRD) characterization to investigate the quality of the flexible YSZ substrate before and after multiple bending (>20 times). During the bending test, the flexible YSZ substrate experienced a high degree of tensile and compressive strain (>1% of strain) and high-temperature annealing (800 °C for 5 min in nitrogen ambient). No peak shifting or broadening was observed from XRD spectra, suggesting excellent mechanical stability of the flexible zirconia substrate. In addition, the energy-dispersive X-ray spectroscopy (EDX) analysis shown in Figure 1c represents the stable chemical composition of flexible YSZ substrate after multiple rounds of thermal annealing. The results also indicate that the flexible YSZ substrate offers good comparability with the conventional CVD system for MoS_2_ growth.

After the completion of a basic thermomechanical test, we performed MoS_2_ growth directly on flexible YSZ substrate. The details of the MoS_2_ growth can be found in reference 42. Briefly, the YSZ substrate was firstly spin-coated with water-soluble solution of ammonium heptamolybdate (AHM) and sodium hydroxide (NaOH). Then the pre-coated YSZ substrate was loaded into the center of a 2-inch tube furnace. In the meantime, 200 mg of S pellets were placed at the downstream and heated up to 210 °C. The temperature was varied from 750 to 850 °C and maintained for 10 min for the growth. Compared to common ammonium thiomolybdate which needs to be dissolved in polar organic solvents, we chose AHM as a precursor since it can dissolve in water, which introduces less contamination [27]. In addition, spin coating of AHM has advantages of speed, simplicity, and requiring no equipment beyond a standard spin coater. During the growth process, AHM is converted to MoO_3_ above 300 °C [41] as the Mo precursor, and reacts with Na to produce sodium molybdate (Na_2_MoO_4_) compounds, followed by MoS_2_ growth on the YSZ substrate after S vapor injection with 500 sccm of N_2_ gas as a carrier gas. Although flexible, YSZ substrate has good thermomechanical properties, the first direct MoS_2_ growth on the flexible YSZ substrate was unsuccessful because of a rough surface. A MoS_2_ growth failure (see Figure S1 in Supplementary Information) is explained both by the secondary electron microscopy (SEM) image and atomic force microscopy (AFM) image of the surface of a flexible YSZ substrate, as shown in Figure 1d,e. The average surface roughness of the bare YSZ substrate was measured to be 25 nm, which was a much higher value than that of the 2D MoS_2_ crystals. Thus, it was difficult to nucleate and continue to grow as a 2D film. To overcome this roughness issue of the YSZ substrate, 50 nm thick SiO_2_ was deposited at 250 °C using a CVD system with a rotation stage. As shown in Figure 1f, the surface roughness of a SiO_2_ flexible YSZ substrate was reduced to 4 nm.

**Figure 2:**
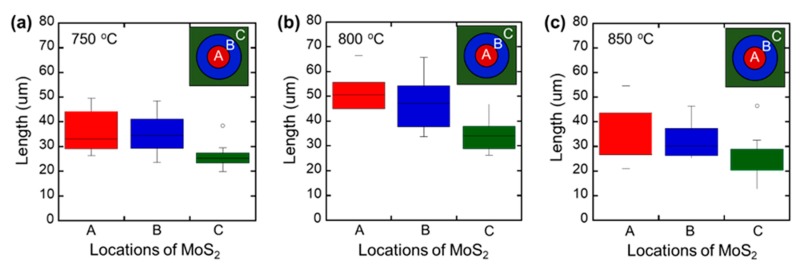
The statistical distribution of the lengths of one side of triangular MoS_2_ crystals grown (**a**) 750 °C, (**b**) 800 °C, and (**c**) 850 °C, respectively. Three different regions, (A), (B), and (C), in each 1.5 cm^2^ size substrate were measured. Region (A) refers to the 3 mm central circle, region (B) refers to another 5 mm circle outside of the region (A), and region (C) refers to the rest of the peripheral area to the edge of the sample.

In addition, the intermediate Na_2_MoO_4_ will react with the SiO_2_ surface to form sodium silicon oxide at high temperatures, so that the wettability of Mo was greatly improved to promote the lateral growth of the MoS_2_ layer compared to the bare YSZ substrate. [42] As shown in Figure 2a–c, few-layered MoS_2_ crystals that were synthesized at different temperatures grew to different sizes. To investigate the size distribution of the MoS_2_ crystals, we divided the 15 mm^2^ size sample into three sections, as follows: the 3-mm area from the edge (labeled as a region [C]), the central 3-mm area (labeled as a region [A]), and the rest of the area between [A] and [C] (labeled as a region [B]). Over 50 MoS_2_ crystals in each zone were measured using a microscope. As seen in Figure 2b, the average length of one side of a triangular MoS_2_ crystal at the center of the samples grown at 800 °C was approximately 50% larger (50.5 μm) than the ones that were grown at 750 °C and 850 °C (33 μm). The largest MoS_2_ crystals exceeded 70 μm and maintained a thickness of less than 5 nm. It should be noted that the size of MoS_2_ crystals at different growth temperatures can be explained by different routes of chemical reactions. At relatively low growth temperatures (750 °C), triangle shape MoS_2_ island formed in a S-rich atmosphere which limits the growth of MoS_2_. As the Mo precursor temperature increases to 800 °C, more unsaturated Mo atoms can be provided and bond with free S atoms in the S-rich environment, leading to the growth of large size MoS_2_ triangular islands. Also, the larger MoS_2_ flakes that were obtained at 800 °C can be explained by a lower sticking coefficient at the temperature higher than 800 °C. The sticking coefficient defines the percentage of precursor that forms stable domains. At 850 °C, the sticking coefficient drops and a significant fraction of precursor will not contribute to deposition due to the enhanced desorption rate at the elevated substrate temperature [43,44]. But when the temperature is increased to 850 °C, the MoS_2_ layers started stacking vigorously and the shape changed to a relatively irregular shape. In Figure 2c, we only counted the size of the triangular MoS_2_ islands.

The size distribution study indicated that the planarized flexible YSZ substrate helped the crystal growth and the average length of one side of the triangular MoS_2_ crystals was similar to the few-layered MoS_2_ crystals that were grown on an SiO_2_/Si substrate. 

## 3. Results and Discussion

**Figure 3:**
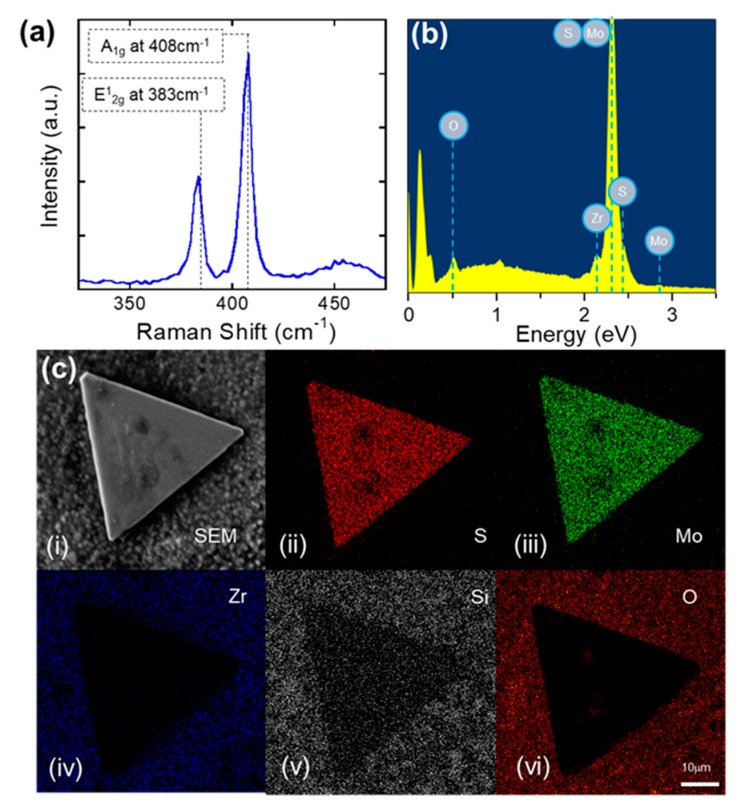
(**a**) Raman spectrum and (**b**) EDX of MoS_2_ crystals grown at 800 °C. (**c**) (i) SEM image, and EDX mapping images of MoS_2_ crystals consists of (ii) S, (iii) Mo, (iv) Zr, (v) Si, and (vi) O atoms from a SiO_2_ coated flexible YSZ substrate.

To investigate the crystal quality of MoS_2_ crystals, Raman spectroscopy was performed using Renishaw Raman spectroscopy. The excitation was provided by a linearly polarized 514 nm excitation laser with a 50× objective lens. The diameter of the laser spot was 1 μm. To avoid MoS_2_ ablation caused by laser-induced heating, all Raman spectra were recorded at the low laser power (200 μW) with an exposure time of 10 s and ten times of accumulations. Figure 3a shows a typical Raman spectrum of a few-layered MoS_2_ crystal on a flexible YSZ substrate. The Raman spectrum clearly presented two Raman-active vibrational modes: an E^1^_2g_ peak and an A^1^_g_ peak. The E^1^_2g_ peak represents the in-plane vibrational mode, whereas the A^1^_g_ peak is the interlayer vibrational mode, and they were observed at around 383 cm^−1^ and 408 cm^−1^, respectively [45]. For the given wavenumber, the distance between E^1^_2g_ and A^1^_g_ peaks increases with the increasing thickness of MoS_2_ because of a thickness-dependent suppression of atomic vibrations by the interlayer van der Waals forces in MoS_2_. Thus, it is possible to determine a thickness of MoS_2_ by measuring the peak positions of the E^1^_2g_ and A^1^_g_ peaks [45,46]. Based on the measured Raman spectrum, the distance in wavenumbers between the E^1^_2g_ and A^1^_g_ peaks was 25 cm^−1^, which yielded ~7 nm (or 9–10 layers of MoS_2_); this is in good agreement with the AFM results in Figure S2 in Supplementary Material. It should be noted that the thickness of the MoS_2_ thin films on a flexible YSZ substrate can be controlled by controlling the loading amount of precursor in our technique. Even thicker films can be prepared by spin-coating the precursor solution with a higher concentration [47]. In addition, the substrate chemistry will also influence the number of layers. Therefore, controlling the SiO_2_ layer quality with the desired amount of –O dangling bond will change the surface energy of the substrate to facilitate heterogeneous nucleation and MoS_2_ growth [48]. Figure 3b–c show a typical SEM and EDX image of a few-layered MoS_2_ crystal. Although morphology of the MoS_2_ crystals that was observed using SEM images was similar to those grown on SiO_2_/Si substrate, EDX was performed to confirm the chemical composition of MoS_2_ crystals using a Hitachi S4000 field emission scanning electron microscope (FESEM). Chemical analysis from the EDS spectrum indicates the presence of Mo and S (elements of MoS_2_), but no other elements such as O (elements of MoO_2_, and MoO_3_) were introduced during the growth. Additionally, elements from a SiO_2_-coated flexible Yttria-stabilized zirconia ceramic (YZS) substrate such as Si, O, and Zr did not diffuse into the MoS_2_ crystals. Quantification of the peaks also showed that the atomic ratio of S to Mo is approximately 2, which is very close to the stoichiometric of ideal MoS_2_ crystals.

**Figure 4:**
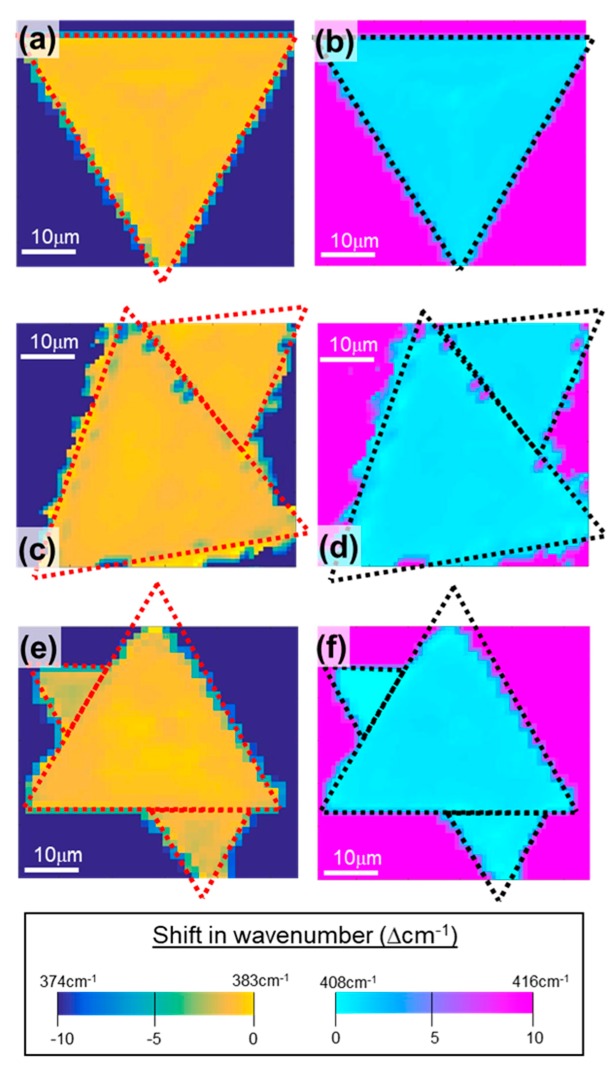
Raman mapping of MoS_2_ crystal showing (left) E^1^_2g_ peak at 383 cm^−1^ and (right) A^1^_g_ peak at 408 cm^−1^ when grown at 750 °C for (**a**,**b**), 800 °C for (**c**,**d**), and 850 °C for (**e**,**f**).

To further investigate the uniformity and chemical composition of MoS_2_ crystals, Raman mapping was performed on three MoS_2_ samples that were grown at 750 °C, 800 °C, or 850 °C (Figure 4a–c). The Raman mapping was conducted at the center of the samples across a 25 × 25 μm^2^ area with 1 μm step increases and a beam spot radius of 0.66 μm under a 100× objective, which consisted of 576 pixels. As shown in Figure 4, the intensity map of the E^1^_2g_ peak (383 cm^−1^ in yellow) and the A^1^_g_ peak (408 cm^−1^ in light blue) of the MoS_2_ crystals show uniformly distributed color, indicating the high homogeneity of the MoS_2_ thickness within the spatial resolution of the Raman instrument (approximately 1 μm). Therefore, combining the perimeter measurement shown in Figure 2, with the Raman mapping results, we confirmed that the SiO_2_ coated flexible YSZ substrate does not influence the quality of MoS_2_ crystal.

**Figure 5:**
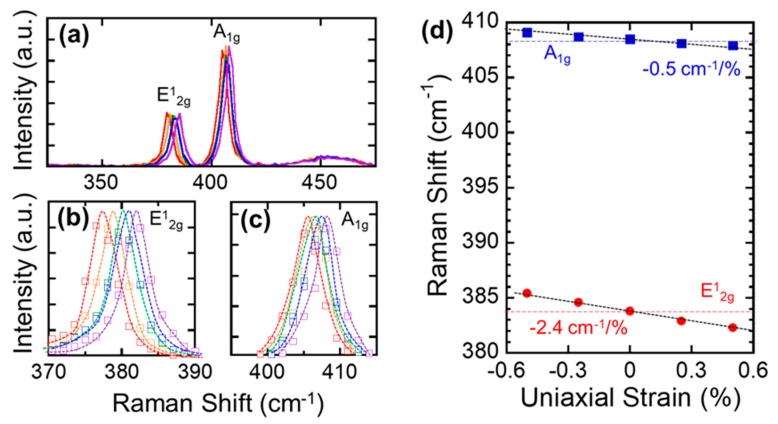
(**a**) Raman spectra of MoS_2_ crystal measured under the uniaxial strains and magnified Raman spectra of (**b**) E^1^_2g_ mode and (**c**) A^1^_g_ mode. Rainbow color lines represent different types and intensities of strains; (red) 0.5% compressive strain, (orange) 0.25% compressive strain, (green) flat, (blue) 0.25% tensile strain, (purple) 0.5% tensile strain. (**d**) E^1^_2g_ mode and A1g mode as a function of applied strain.

Finally, we investigated the evolution of the MoS_2_ Raman spectra under different uniaxial strain conditions. Raman spectroscopy has been widely used to investigate strain in other 2D materials, because of its nondestructive characterization capability of lattice vibrations sensitive to strains [49,50,51,52]. The thickness of MoS_2_ for this stain-Raman spectrum study was the same as for the MoS_2_ crystals that were used for the Raman characterization. To accurately measure the changes in the Raman spectra under the uniaxial strain conditions, we employed convex- and concave-shaped metal molds that had different curve radii ranging from 25 mm to 50 mm, which corresponded to the uniaxial tensile and compressive strains up to 0.5%. The amount of strain applied to the MoS_2_ is calculated by the following equation:

strain (%) = 1/[(2R/ΔR) + 1]

where R is the bending radius and ΔR is a thickness of bended object including a substrate and MoS_2_. The Strain-dependent characteristics of the E^1^_2g_ and A^1^_g_ modes are shown in Figure 5. Figure 5a shows the Raman spectra measured from 0.5% of tensile strain to 0.5% of compressive strain. It should be noted that, in Figure 5d, a negative and a positive strain values denote a tensile and a compressive strain, respectively. Figure 5b,c are the magnified spectra of the E1_2g_ and A^1^_g_ peaks under different strain conditions: red and orange indicate 0.5% and 0.25% tensile strain, respectively, while blue and purple indicate 0.25% and 0.5% compressive strain, respectively. As shown in Figure 5d, which presents the position of the E^1^_2g_ and A^1^_g_ peaks as a function of strain, we observed a very small shift of the A^1^_g_ mode at a rate of −0.5 cm^−1^/% strain. The E^1^_2g_ mode, however, showed a considerably larger shift, at a rate of −2.4 cm^−1^/%. These rates are slightly higher than the rates that were observed when using a PDMS substrate, and they were similar to the results from the freestanding MoS_2_ crystals [41,53]. This higher rate of E^1^_2g_ mode can be explained by stronger chemical bonds between MoS_2_ and YSZ substrate. It is known that the E^1^_2g_ mode is responsible for opposite vibration of two S atoms with respect to the Mo atom in the basal plane, while the A^1^_g_ mode results from the vertical or out-of plane vibration of S atoms in opposite directions [41]. Our result clearly indicates that the dominated covalent bonding between Mo and S atoms is fairly sensitive to the in-plane uniaxial strain. In addition, it is suggested that MoS_2_ crystals were strongly bonded to the flexible zirconia substrate without experiencing any mechanical slip or layer sliding. For applied strain in the range of −0.5% to +0.5%, the peak shifting rate of the E^1^_2g_ and A^1^_g_ peaks was nearly constant and did not exhibit any hysteresis in multiple loading/unloading strain cycles (Figure S3 in Supplementary Material). This indicates that the strain does not generate a significant number of defects in the MoS_2_ crystals.

## 4. Conclusions

In conclusion, high-quality few-layered MoS_2_ crystals were successfully grown on a flexible ZrO_2_ substrate. To planarize the rough ZrO_2_ substrate, 50 nm of SiO_2_ was deposited before the growth. MoS_2_ crystals grown at 800 °C showed a uniform and large crystal size. The EDX and Raman mapping revealed a good uniformity of MoS_2_ crystals with a stable chemical composition of one Mo and two S. The Raman characterization under bending conditions also exhibited a stable material property that was consistent with other results. ZrO_2_ crystals and other 2D materials can be directly grown on a flexible ZrO_2_ substrate, and therefore, we suggest that the direct growth of a 2D materials on a flexible substrate offers a new opportunity to enable the use of 2D materials on a larger substrate at a lower cost. This would provide high performance and flexible electronic/optoelectronic applications without sacrificing the intrinsic performance of 2D materials.

## Supplementary Materials

The following are available online at https://www.mdpi.com/2079-4991/9/10/1456/s1, Figure S1: Microscopic images of MoS_2_ taken from YSZ substrate under different temperature from 700 °C to 800 °C. Figure S2: AFM image of few-layered MoS_2_ crystal on flexible YSZ substrate. Figure S3: Raman shift rate of E1_2g_ and A^1^_g_ mode peaks as a function of bending cycles.

## References

[1] Kim K.S., Zhao Y., Jang H., Lee S.Y., Kim J.M., Kim K.S., Ahn J.-H., Kim P., Choi J.-Y., Hong B.H. (2009). Large-scale pattern growth of graphene films for stretchable transparent electrodes. Nature.

[2] Tao L., Cinquanta E., Chiappe D., Grazianetti C., Fanciulli M., Dubey M., Molle A., Akinwande D. (2015). Silicene field-effect transistors operating at room temperature. Nat. Nanotechnol..

[3] Liang S., Hasan M.N., Seo J.-H. (2019). Direct Observation of Raman Spectra in Black Phosphorus under Uniaxial Strain Conditions. Nanomaterials.

[4] Ni Z., Liu Q., Tang K., Zheng J., Zhou J., Qin R., Gao Z., Yu D., Lu J. (2011). Tunable bandgap in silicene and germanene. Nano Lett..

[5] Peng X., Peng L., Wu C., Xie Y. (2014). Two dimensional nanomaterials for flexible supercapacitors. Chem. Soc. Rev..

[6] Liu H., Neal A.T., Zhu Z., Luo Z., Xu X., Tománek D., Ye P.D. (2014). Phosphorene: An unexplored 2D semiconductor with a high hole mobility. ACS Nano.

[7] Manzeli S., Ovchinnikov D., Pasquier D., Yazyev O.V., Kis A. (2017). 2D transition metal dichalcogenides. Nat. Rev. Mater..

[8] Liu H., Du Y., Deng Y., Peide D.Y. (2015). Semiconducting black phosphorus: Synthesis, transport properties and electronic applications. Chem. Soc. Rev..

[9] Wang F., Seo J.-H., Luo G., Starr M.B., Li Z., Geng D., Yin X., Wang S., Fraser D.G., Morgan D. (2016). Nanometre-thick single-crystalline nanosheets grown at the water–air interface. Nat. Commun..

[10] Thi Q.H., Kim H., Zhao J., Ly T.H. (2018). Coating two-dimensional MoS2 with polymer creates a corrosive non-uniform interface. NPJ 2D Mater. Appl..

[11] Akinwande D., Brennan C.J., Bunch J.S., Egberts P., Felts J.R., Gao H., Huang R., Kim J.-S., Li T., Li Y. (2017). A review on mechanics and mechanical properties of 2D materials—Graphene and beyond. Extreme Mechan. Lett..

[12] Frank I., Tanenbaum D.M., van der Zande A.M., McEuen P.L. (2007). Mechanical properties of suspended graphene sheets. J. Vac. Sci. Technol. B Microelectron. Nanometer Struct..

[13] Scarpa F., Adhikari S., Phani A.S. (2009). Effective elastic mechanical properties of single layer graphene sheets. Nanotechnology.

[14] Kim J., Baik S.S., Ryu S.H., Sohn Y., Park S., Park B.-G., Denlinger J., Yi Y., Choi H.J., Kim K.S. (2015). Observation of tunable band gap and anisotropic Dirac semimetal state in black phosphorus. Science.

[15] Castellanos-Gomez A. (2015). Black Phosphorus: Narrow Gap, Wide Applications. J. Phys. Chem. Lett..

[16] Gao L. (2017). Flexible device applications of 2D semiconductors. Small.

[17] Akinwande D., Petrone N., Hone J. (2014). Two-dimensional flexible nanoelectronics. Nat. Commun..

[18] Park D.-W., Schendel A.A., Mikael S., Brodnick S.K., Richner T.J., Ness J.P., Hayat M.R., Atry F., Frye S.T., Pashaie R. (2014). Graphene-based carbon-layered electrode array technology for neural imaging and optogenetic applications. Nat. Commun..

[19] Cheng R., Jiang S., Chen Y., Liu Y., Weiss N., Cheng H.-C., Wu H., Huang Y., Duan X. (2014). Few-layer molybdenum disulfide transistors and circuits for high-speed flexible electronics. Nat. Commun..

[20] Wu Y., Zou X., Sun M., Cao Z., Wang X., Huo S., Zhou J., Yang Y., Yu X., Kong Y. (2016). 200 GHz Maximum Oscillation Frequency in CVD Graphene Radio Frequency Transistors. ACS Appl. Mater. Interfaces.

[21] Yeh C.-H., Lain Y.-W., Chiu Y.-C., Liao C.-H., Moyano D.R., Hsu S.S.H., Chiu P.-W. (2014). Gigahertz Flexible Graphene Transistors for Microwave Integrated Circuits. ACS Nano.

[22] Salvatore G.A., Münzenrieder N., Barraud C., Petti L., Zysset C., Büthe L., Ensslin K., Tröster G. (2013). Fabrication and Transfer of Flexible Few-Layers MoS2 Thin Film Transistors to Any Arbitrary Substrate. ACS Nano.

[23] Das S., Chen H.-Y., Penumatcha A.V., Appenzeller J. (2012). High performance multilayer MoS2 transistors with scandium contacts. Nano Lett..

[24] Berciaud S., Ryu S., Brus L.E., Heinz T.F. (2008). Probing the intrinsic properties of exfoliated graphene: Raman spectroscopy of free-standing monolayers. Nano Lett..

[25] Xie J., Zhang J., Li S., Grote F., Zhang X., Zhang H., Wang R., Lei Y., Pan B., Xie Y. (2013). Controllable disorder engineering in oxygen-incorporated MoS_2_ ultrathin nanosheets for efficient hydrogen evolution. J. Am. Chem. Soc..

[26] Mannix A.J., Kiraly B., Hersam M.C., Guisinger N.P. (2017). Synthesis and chemistry of elemental 2D materials. Nat. Rev. Chem..

[27] Shi Y., Li H., Li L.J. (2015). Recent advances in controlled synthesis of two-dimensional transition metal dichalcogenides via vapour deposition techniques. Chem. Soc. Rev..

[28] Zhan Y., Liu Z., Najmaei S., Ajayan P.M., Lou J. (2012). Large-area vapor-phase growth and characterization of MoS_2_ atomic layers on a SiO_2_ substrate. Small.

[29] Lin Y.C., Zhang W., Huang J.K., Liu K.K., Lee Y.H., Liang C.T., Chu C.W., Li L.J. (2012). Wafer-scale MoS_2_ thin layers prepared by MoO_3_ sulfurization. Nanoscale.

[30] Vangelista S., Cinquanta E., Martella C., Alia M., Longo M., Lamperti A., Mantovan R., Basset F.B., Pezzoli F., Molle A. (2016). Towards a uniform and large-scale deposition of MoS_2_ nanosheets via sulfurization of ultra-thin Mo-based solid films. Nanotechnology.

[31] Kang K., Xie S., Huang L., Han Y., Huang P.Y., Mak K.F., Kim C.J., Muller D., Park J. (2015). High-mobility three-atom-thick semiconducting films with wafer-scale homogeneity. Nature.

[32] Van Der Zande A.M., Huang P.Y., Chenet D.A., Berkelbach T.C., You Y., Lee G.H., Heinz T.F., Reichman D.R., Muller D.A., Hone J.C. (2013). Grains and grain boundaries in highly crystalline monolayer molybdenum disulphide. Nat. Mater..

[33] Najmaei S., Liu Z., Zhou W., Zou X., Shi G., Lei S., Yakobson B.I., Idrobo J.C., Ajayan P.M., Lou J. (2013). Vapour phase growth and grain boundary structure of molybdenum disulphide atomic layers. Nat. Mater..

[34] Yu Y., Li C., Liu Y., Su L., Zhang Y., Cao L. (2013). Controlled scalable synthesis of uniform, high-quality monolayer and few-layer MoS_2_ films. Sci. Rep..

[35] Lin Z., Zhao Y., Zhou C., Zhong R., Wang X., Tsang Y.H., Chai Y. (2015). Controllable growth of large–size crystalline MoS_2_ and resist-free transfer assisted with a Cu thin film. Sci. Rep..

[36] Urban F., Martucciello N., Peters L., McEvoy N., Bartolomeo A.D. (2018). Environmental effects on the electrical characteristics of back-gated WSe_2_ field-effect transistors. Nanomaterials.

[37] Barin G.B., Song Y., Gimenez I.F., Filho A.G.S., Barreto L.S., Kong J. (2015). Optimized graphene transfer: Influence of polymethylmethacrylate (PMMA) layer concentration and baking time on graphene final performance. Carbon.

[38] Ahn Y., Kim H., Kim Y.H., Yi Y., Kim S.I. (2013). Procedure of removing polymer residues and its influences on electronic and structural characteristics of graphene. Appl. Phys. Lett..

[39] Pirkle A., Chan J., Venugopal A., Hinojos D., Magnuson C.W., McDonnell S., Colombo L., Vogel E.M., Ruoff R.S., Wallace R.M. (2011). The effect of chemical residues on the physical and electrical properties of chemical vapor deposited graphene transferred to SiO_2_. Appl. Phys. Lett..

[40] Brandon J., Taylor R. (1989). Thermal properties of ceria and yttria partially stabilized zirconia thermal barrier coatings. Surf. Coat. Technol..

[41] Wang Y., Cong C., Qiu C., Yu T. (2013). Raman spectroscopy study of lattice vibration and crystallographic orientation of monolayer MoS_2_ under uniaxial strain. Small.

[42] Kim H., Han G.H., Yun S.J., Zhao J., Keum D.H., Jeong H.Y., Ly T.H., Jin Y., Park J.-H., Moon B.H. (2017). Role of alkali metal promoter in enhancing lateral growth of monolayer transition metal dichalcogenides. Nanotechnology.

[43] Withanage S.S., Kalita H., Chung H.S., Roy T., Jung Y., Khondaker S.I. (2018). Uniform Vapor-Pressure-Based Chemical Vapor Deposition Growth of MoS_2_ Using MoO_3_ Thin Film as a Precursor for Coevaporation. ACS Omega.

[44] Yue R., Nie Y., Walsh L.A., Addou R., Liang C., Lu N., Barton A.T., Zhu H., Che Z., Barrera D. (2017). Nucleation and growth of WSe_2_: Enabling large grain transition metal dichalcogenides. 2D Mater..

[45] Li H., Zhang Q., Yap C.C.R., Tay B.K., Edwin T.H.T., Olivier A., Baillargeat D. (2012). From bulk to monolayer MoS2: Evolution of Raman scattering. Adv. Funct. Mater..

[46] Chakraborty B., Matte H.R., Sood A., Rao C. (2013). Layer-dependent resonant Raman scattering of a few layer MoS_2_. J. Raman Spectrosc..

[47] Yang H., Giri A., Moon S., Shin S., Myoung J.M., Jeong U. (2017). Highly scalable synthesis of MoS_2_ thin films with precise thickness control via polymer-assisted deposition. Chem. Mater..

[48] Jeon J., Jang S.K., Jeon S.M., Yoo G., Jang Y.H., Park J.H., Lee S. (2015). Layer-controlled CVD growth of large-area two-dimensional MoS_2_ films. Nanoscale.

[49] Mikael S., Seo J.-H., Park D.-W., Kim M., Mi H., Javadi A., Gong S., Ma Z. (2017). Triaxial compressive strain in bilayer graphene enabled by nitride stressor layer. Extreme Mechan. Lett..

[50] Mikael S., Seo J.-H., Javadi A., Gong S., Ma Z. (2016). Wrinkled bilayer graphene with wafer scale mechanical strain. Appl. Phys. Lett..

[51] Mi H., Mikael S., Liu C.-C., Seo J.-H., Gui G., Ma A.L., Nealey P.F., Ma Z. (2015). Creating periodic local strain in monolayer graphene with nanopillars patterned by self-assembled block copolymer. Appl. Phys. Lett..

[52] Kim M., Mi H., Cho M., Seo J.-H., Zhou W., Gong S., Ma Z. (2015). Tunable biaxial in-plane compressive strain in a Si nanomembrane transferred on a polyimide film. Appl. Phys. Lett..

[53] Liu Z., Amani M., Najmaei S., Xu Q., Zou X., Zhou W., Yu T., Qiu C., Birdwell A.G., Crowne F.J. (2014). Strain and structure heterogeneity in MoS2 atomic layers grown by chemical vapour deposition. Nat. Commun..

