# Supplementary material for: Direct Growth of Two Dimensional Molybdenum Disulfide on Flexible Ceramic Substrate
Source: Nanomaterials (Basel). 2019 Oct 14;9(10):1456. doi: 10.3390/nano9101456 (PMC6835219; doi:10.3390/nano9101456)
Supplement: Supplementary file 1 [file nanomaterials-09-01456-s001.pdf]

nanomaterials

Supplementary Material

Direct Growth of Two Dimensional Molybdenum Disulfide on Flexible Ceramic Substrate

Yixiong Zheng[1,†], Chunyan Yuan[1,†], Sichen Wei[1], Hyun Kim[2], Fei Yao[1,*], Jung-Hun Seo[1,*]

[1] Department of Materials Design and Innovation, University at Buffalo, Buffalo, NY 14260 USA
[2] Component Solution Business Unit, Samsung Electro-Mechanics, Suwon, 16674, Korea
* Correspondence: feiyao@buffalo.edu, junghuns@buffalo,edu

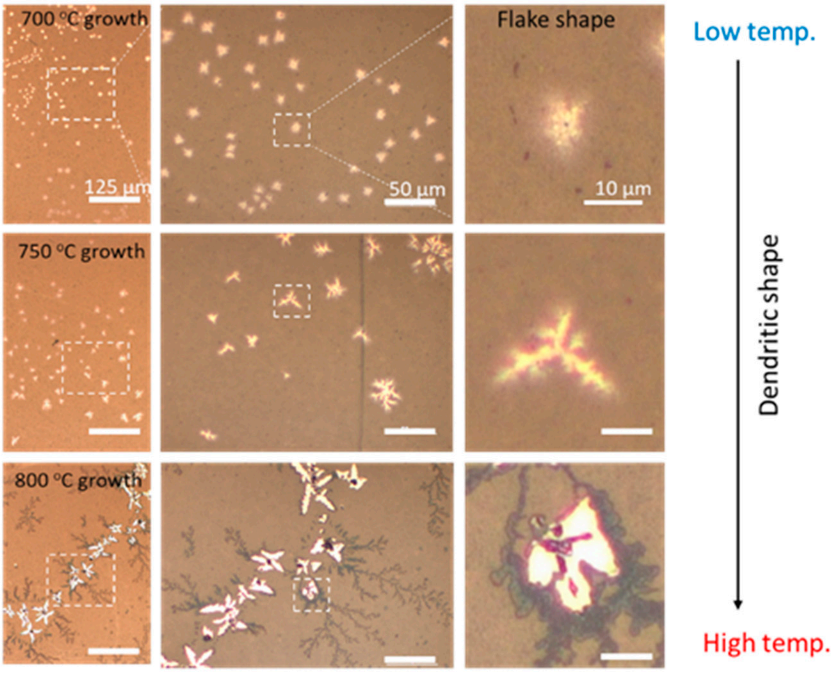

Figure S1. Microscopic images of MoS_2 taken from YSZ substrate under different temperature from 700 °C to 800 °C showing the formation of dendritic shape MoS_2 crystals. The failure of triangular shaped MoS_2 growth was caused by a rough YSZ surface, namely, deep valleys in bare YSZ substrate prevents horizontal nucleation to grow MoS_2 as a 2D format, regardless of growth temperature.

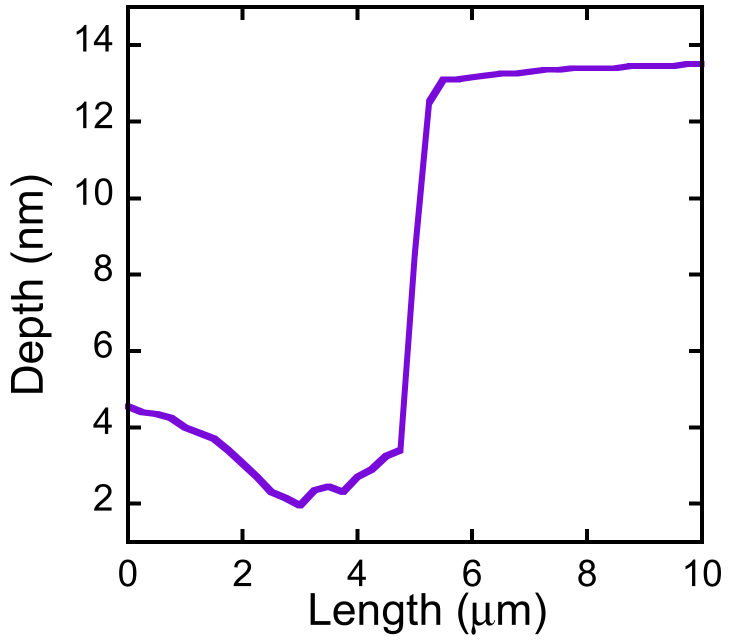

Figure S2. AFM image of few-layered MoS_2 crystal on flexible YSZ substrate to show the height of the typical MoS_2 crystal.

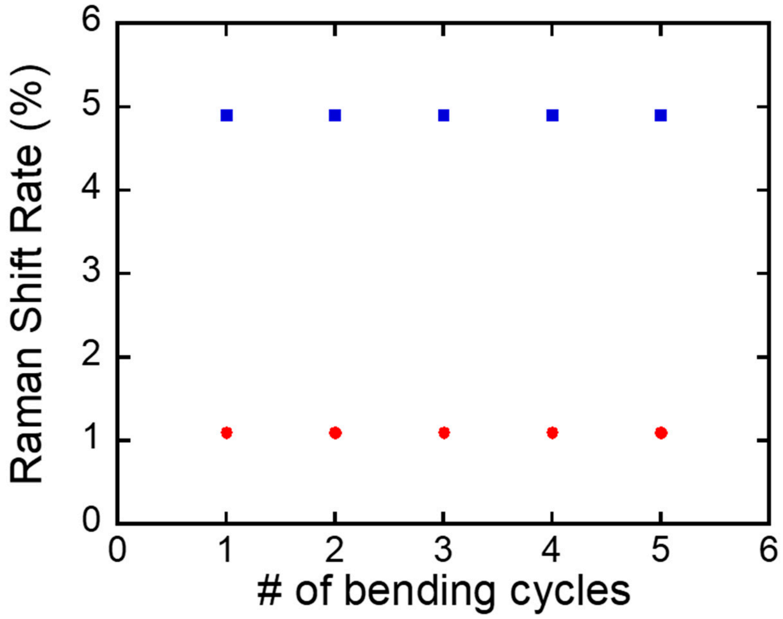

Figure S3. Raman shift rate of E^1_{2g} and A^1_g mode peaks as a function of bending cycles up to 5 times, showing a stable shifting rate.
